# Optogenetic Study of Anterior BNST and Basomedial Amygdala Projections to the Ventromedial Hypothalamus

**DOI:** 10.1523/ENEURO.0204-18.2018

**Published:** 2018-07-02

**Authors:** Ryo Yamamoto, Nowrin Ahmed, Tetsufumi Ito, Nur Zeynep Gungor, Denis Pare

**Affiliations:** 1 Department of Physiology, Kanazawa Medical University, Ishikawa 920-0293, Japan; 2 Center for Molecular and Behavioral Neuroscience, Rutgers University-Newark, 197 University Avenue, Newark, NJ; 3 Department of Anatomy, Kanazawa Medical University, Ishikawa 920-0293, Japan; 4 RIKEN Center for Brain Science 2-1 Hirosawa, Wako-shi, Saitama 351-0198, Japan

**Keywords:** Amygdala, anxiety, BNST, defensive behaviors, fear, ventromedial hypothalamus

## Abstract

The basomedial amygdala (BM) influences the ventromedial nucleus of the hypothalamus (VMH) through direct glutamatergic projections as well as indirectly, through the anterior part of the bed nucleus of the stria terminalis (BNSTa). However, BM and BNSTa axons end in a segregated fashion in VMH. BM projects to the core of VMH, where VMH’s projection cells are located, whereas BNSTa projects to the shell of VMH, where GABAergic cells that inhibit core neurons are concentrated. However, the consequences of this dual regulation of VMH by BM and BNSTa are unknown. To study this question, we recorded the responses of VMH’s shell and core neurons to the optogenetic activation of BM or BNSTa inputs in transgenic mice that selectively express Cre-recombinase in glutamatergic or GABAergic neurons. Glutamatergic BM inputs fired most core neurons but elicited no response in GABAergic shell neurons. Following BM infusions of AAV-EF1α-DIO-hChR2-mCherry in Vgat-ires-Cre-Ai6 mice, no anterograde labeling was observed in the VMH, suggesting that GABAergic BM neurons do not project to the VMH. In contrast, BNSTa sent mostly GABAergic projections that inhibited both shell and core neurons. However, BNSTa-evoked IPSPs had a higher amplitude in shell neurons. Since we also found that activation of GABAergic shell neurons causes an inhibition of core neurons, these results suggest that depending on the firing rate of shell neurons, BNSTa inputs could elicit a net inhibition or disinhibition of core neurons. Thus, the dual regulation of VMH by BM and BNSTa imparts flexibility to this regulator of defensive and social behaviors.

## Significance Statement

The ventromedial hypothalamus (VMH), a critical component of the innate defense network, is regulated by the basomedial amygdala (BM), which supplies non-olfactory information to the VMH, and BNST, another structure mediating defensive behaviors and a recipient of BM inputs. BM projects to the core of VMH, where its projection cells are located, whereas BNST projects to the shell of VMH, where GABAergic cells that inhibit core neurons are concentrated. However, the consequences of this dual regulation of VMH by BM and BNST are unknown. Our results indicate that, depending on the firing rate of shell neurons, the influence of BNST can shift from an inhibition to a disinhibition of core neurons, thus imparting flexibility to this innate defensive network.

## 

The ventromedial hypothalamic nucleus (VMH) is a critical component of the brain’s innate defense network (Fernandez De Molina and Hunsperger, 1962; Dielenberg et al., 2001; Martinez et al., 2008; Kunwar et al., 2015) and a regulator of various social behaviors (Pfaff and Sakuma, 1979a,b; Yang et al., 2013; Lee et al., 2014; Ishii et al., 2017; Hashikawa et al., 2017). Depending on the modality, different pathways relay sensory information to the VMH. A major route for the transfer of olfactory (volatile and pheromone) information to the VMH involves the medial amygdala and posterior region of the bed nucleus of the stria terminalis (BNSTp; Canteras et al., 1994; Dong and Swanson, 2004; Hong et al., 2014; Hashikawa et al., 2016; Padilla et al., 2016; Hashikawa et al., 2017). In contrast, auditory and visual information about predators and conspecifics are thought to reach the VMH via the basomedial nucleus of the amygdala (BM; McDonald et al., 1999; Martinez et al., 2011; Gross and Canteras, 2012).

**Figure 1.**
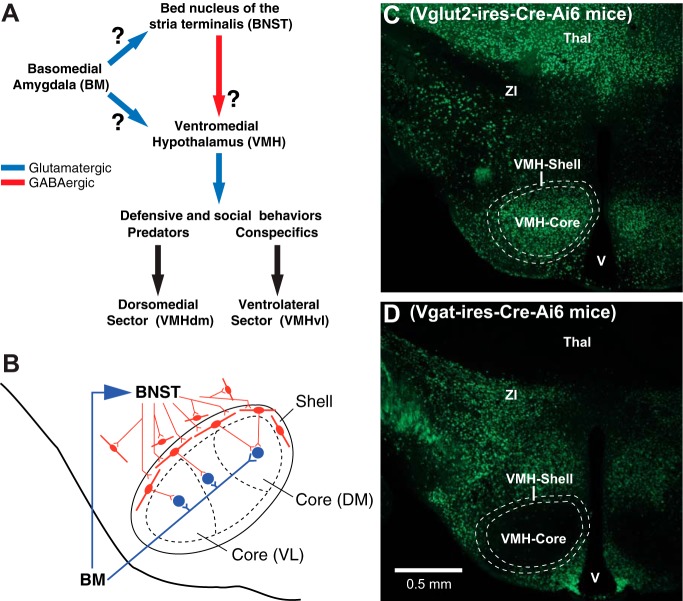
Network investigated in the present study. ***A***, Summary of connections investigated. Blue and red lines indicate glutamatergic and GABAergic connections, respectively. The basomedial nucleus of the amygdala (BM) sends parallel projections to the ventromedial hypothalamic nucleus (VMH) and to the anterior portion of the bed nucleus of the stria terminalis (BNSTa). In turn, BNSTa sends projections to the VMH, which has been implicated in the regulation of defensive and social behaviors. Because prior studies have reported that most extrinsic projections of BM and BNST respectively originate from glutamatergic and GABAergic neurons, arrows making these connections are color-coded accordingly. However, this remains to be established, hence the question marks. ***B***, BM and BNSTa send non-overlapping projections to the VMH. BM projects to the core of the VMH, where projection cells are located, whereas BNSTa projects to the shell of VMH and surrounding region, where GABAergic cells are found. The core of VMH is divided in sectors (DM, dorsomedial; VL, ventrolateral). It should be noted that the shell also contains a small contingent of glutamatergic neurons. However, in contrast to the GABA cells, which are homogeneously distributed in the shell, the glutamatergic cells occur in small but dense clusters that correspond to the “cell bridges” described in Fig. 2. The properties of these glutamatergic neurons are not investigated in the present study. ***C***, Distribution of glutamatergic cells in Vglut2-Cre-IRES-knock-in mice crossed with Ai6 reporter mice. ***D***, Distribution of GABAergic cells in Vgat-Cre-IRES-knock-in mice crossed with Ai6 reporter mice. Thal, thalamus; V, ventricle; ZI, zona incerta.

However, the regulation of the VMH by BM is complex (Fig. 1*A*). Indeed, besides projecting to the VMH (Petrovich et al., 1996), BM also influences it indirectly, through neurons in the anterior part of the bed nucleus of the stria terminalis (BNSTa; Krettek and Price, 1978; Dong et al., 2001). Like the VMH, BNSTa has been implicated in the genesis of defensive behaviors, particularly anxiety-like states with ill-defined and unpredictable triggers (Walker et al., 2009). However, unlike BNSTp, which contains many glutamatergic neurons, the vast majority of BNSTa cells are GABAergic (Day et al., 1999; Poulin et al., 2009) such that when BM recruits BNSTa, its targets should be inhibited. Complicating matters further, BM and BNSTa send non-overlapping projections to the VMH (Fig. 1*B*). Indeed, the VMH is comprised of two sectors: a core region that contains the nucleus’s glutamatergic projection cells, and a cell-poor shell region that surrounds the core and is mostly populated by GABAergic cells, which are thought to inhibit core neurons (Murphy and Renaud, 1968; Millhouse, 1973a,b; Fu and van den Pol, 2008).

Because BM inputs are confined to the core of VMH (Petrovich et al., 1996) whereas BNSTa axons end in the shell and surrounding area (Dong and Swanson, 2006), it is possible that BM and BNSTa synergistically excite VMH’s projection neurons, the former through a direct synaptic excitation, and the latter through disinhibition. At odds with this possibility, however, the distal dendrites of VMH’s core neurons extend into the shell and beyond (Millhouse, 1973a,b; Fu and van den Pol, 2008; Griffin and Flanagan-Cato, 2009). Consequently, they might also receive direct inhibitory inputs from BNSTa.

Thus, the present study was undertaken to shed light on the impact of BM and BNSTa inputs on the VMH. To this end, we performed whole-cell patch recordings of shell and core VMH neurons and, in separate experiments, optogenetically activated glutamatergic BM or GABAergic BNSTa inputs to the VMH. Our results indicate that depending on the firing rate of shell neurons, the influence of BNSTa can shift from an inhibition to a disinhibition of core neurons.

## Materials and Methods

### Animals and virus injections

All procedures were approved by the Institutional Animal Care and Use Committees of Rutgers University and Kanazawa Medical University. To visualize GABAergic or glutamatergic VMH neurons, we crossed Ai6 reporter mice (Stock 007906) with Vgat-ires-Cre knock-in mice (Stock 016962) or Vglut2-ires-Cre mice (Stock 016963), respectively. In keeping with prior reports (Vong et al., 2011), sections from the Vglut2-ires-Cre-Ai6 (Fig. 1*C*) and Vgat-ires-Cre-Ai6 (Fig. 1*D*) mice looked like negatives of each other, and the expression of the fluorescent reporter ZsGreen1 matched prior observations regarding the location of glutamatergic and GABAergic neurons in the brain (Poulin et al., 2009).

The Cre-dependent expression of the excitatory opsin Channelrhodopsin (ChR2) was restricted to GABAergic or glutamatergic neurons by infusing the virus AAV-EF1α-DIO-hChR2-mCherry (UPenn Vector Core) at the origin of VMH inputs (BM or BNSTa) in Vgat-ires-Cre-Ai6 or Vglut2-ires-Cre-Ai6 mice, respectively. To this end, male or female mice (2–3 months old) were anesthetized with a mixture of isoflurane and oxygen and placed into a stereotaxic apparatus. Their body temperature was kept at ∼37°C. Atropine methyl nitrate (0.05 mg/kg, i.m.) was administered to aid breathing. Betadine and alcohol were used to clean the scalp. Bupivacaine was injected in the region to be incised (0.125% solution, s.c.). Small burr holes were drilled above BNSTa (in mm, relative to bregma: AP, 0.2; ML, 0.8; DV, 3.9), BM (AP, 2.2; ML, 2.9; DV, 4.8), or VMH (AP, 1.3; ML, 0.7; DV, 5.5). Nanoject II (Drummond Scientific Co.) was used to make pressure injections of the virus (50 nl for BNSTa and hypothalamus; 100 nl for BM) at a rate of 9.6 nL/5 s using glass pipettes pulled to an outer tip diameter of ∼70 µm using a PE-22 puller (Narishige Instruments).

At the conclusion of the infusion, the scalp was sutured, a local antibiotic (Neosporin paste) was applied to the wound, and an analgesic was administered (Ketoprofen, 2 mg/kg, s.c. daily for 3 days). Mice were used for *in vitro* whole-cell recording experiments ∼3 weeks after the virus infusions.

### Slice preparation

Mice were deeply anesthetized with isoflurane. After abolition of reflexes, they were perfused transcardially with an ice-cold solution containing (in mm) 103 NMDG, 2.5 KCl, 10 MgSO_4_, 30 NaHCO_3_, 1.2 NaH_2_PO_4_, 0.5 CaCl_2_, 25 glucose, 20 HEPES, 2 thiourea, 3 Na-pyruvate, and 12 *N*-acetyl-L-cysteine. The brains were sectioned using a vibrating microtome at a thickness of 300 µm while submerged in the above solution. Subsequently, slices were kept submerged in the oxygenated solution containing (in mm) 126 NaCl, 2.5 KCl, 1 MgCl_2_, 26 NaHCO_3_, 1.25 NaH_2_PO_4_, 2 CaCl_2_, and 10 glucose (pH 7.3, 300 mOsm). The holding chamber was kept at 34°C for 5 min and then returned to room temperature. 1 h later, a first slice was transferred to the recording bath, which was perfused with the same oxygenated solution at 32°C (6 ml/min).

### Electrophysiology

Whole-cell recordings of shell or core VMH neurons were obtained under visual guidance using infrared differential interface contrast microscopy. We used pipettes pulled from borosilicate glass capillaries (resistance 5–8 MΩ). The intracellular solution contained (in mm): 130 K-gluconate, 10 HEPES, 10 KCl, 2 MgCl_2_, 2 ATP-Mg, and 0.2 GTP-Tris (hydroxymethyl)aminomethane, pH 7.2, 280 mOsm. The liquid junction potential was 10 mV with this solution. However, the membrane potential (*V_m_*) values listed below were not corrected for the junction potential. We used a MultiClamp 700B Amplifier (Molecular Devices) and digitized the data at 20 kHz with a Digidata-1550 interface controlled by pClamp-10.3 (Molecular Devices).

To characterize the electroresponsive properties of the cells, we applied graded series of current pulses (±10-pA increments; 500 ms; 0.2 Hz) from rest as well as more negative and positive membrane potentials, as determined by DC current injection. The input resistance of the cells was calculated from their voltage response to –20-pA current injections. To activate ChR2-expressing axons, blue light stimuli (2 ms) were applied at 0.05 or 5 Hz through an optic fiber (200–300 µm) patch cable coupled to a PlexBright tabletop blue LED module (Plexon). The power density at the fiber tips was ∼700 mW/mm^2^. The distance between the fiber optic tip and recording pipette was adjusted to ∼200 µm. Postsynaptic potentials or currents were evoked from several membrane potentials. The IPSP or IPSC reversal potentials were calculated from the linear fit of fluctuations in IPSP or IPSC amplitudes as a function of membrane potential.

### Identification of the core and shell regions of VMH

**Figure 2.**
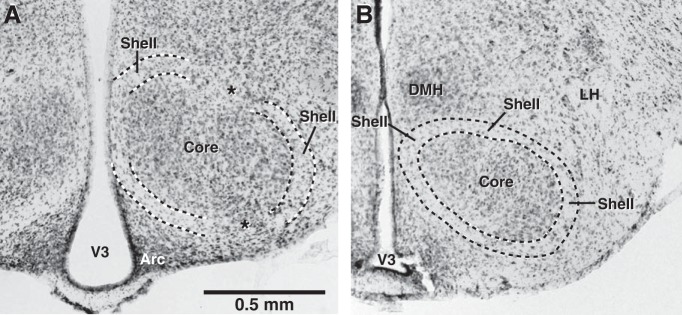
Histologic features of the VMH shell and core regions. Two coronal sections stained with cresyl violet. Whereas the core region is characterized by a high cell density, the shell region is sparsely populated with neurons and appears as a ring of fibers that surrounded the core. In some places, the shell is interrupted by cell bridges (asterisks in ***A***). No recordings were obtained from such ambiguous regions. Arc, arcuate hypothalamic nucleus; DMH, dorsomedial hypothalamic nucleus; LH, lateral hypothalamic area; V3, third ventricle.

To identify the borders of the VMH shell and core regions, we relied on the following criteria. First, using infrared differential interference contrast optics, the shell region appeared as a conspicuous ring of fibers, 60–100 µm in width, which surrounded the core (Fig. 2). Second, the shell was sparsely populated with neurons, whereas the core had a high cell density (Fig. 2). Third, when working with Vglut2-ires-Cre-Ai6 mice, the shell/core border coincided with a marked increase in the number of reporter-positive neurons from the shell to the core region. In contrast, when working with Vgat-ires-Cre-Ai6 mice, the shell/core border coincided with a clear drop in the number of reporter-positive cells, from the shell to the core region. Note that depending on the exact antero-posterior level, the relative size of the shell and core varied slightly. Also, in some instances, the ring of fibers surrounding the core was interrupted by cell bridges (asterisks in Fig. 2*A*). Recordings were obtained only when all the above criteria were met, but not in the ambiguous regions.

### Microscopic observations

Before the recordings, we ascertained that the virus infusions had reached their intended target using fluorescence microscopy (Zeiss, Axioscope). A more detailed examination of the infusion sites was performed after the experiments. To this end, slices were fixed in 4% paraformaldehyde for 12 hours and then examined with Stereo Investigator v11 (MBF Biosciences) and Nikon Eclipse E800. The boundaries of BNSTa and BM were drawn on the bright-field images, and the fluorescence images were superimposed on the bright-field images to assess virus diffusion. All the data described below were obtained in mice where the virus infusion site (BM or BNSTa) was centered on the intended target and no infected neurons could be detected in adjacent structures.

Using Vglut2-ires-Cre-Ai6 and Vgat-ires-Cre-Ai6 mice, we assessed the relative density of Vgat^+^ and Vglut2^+^ neurons in coronal sections. Five coronal sections from one mouse of each type were used for this purpose. Confocal images of the VMH region were taken using Olympus Fluoview FV1000, and the position of the ZsGreen1 positive cells was mapped. Next, the sections were counterstained with cresyl violet to reveal the borders of the shell and core regions. The fluorescence images were then placed in register with the photographs of the counterstained sections, and the labeled cells were counted separately in the two VMH regions. Counts of glutamatergic and GABAergic cell counts obtained from sections at the same antero-posterior levels were used to compute ratios of the two cell types.

### Morphology

To study the morphology of recorded neurons, in a subset of experiments, 0.75% biocytin was added to the pipette solution. Biocytin diffused into the cells as their electroresponsive properties were recorded. After termination of the recordings, the slice was removed from the chamber and fixed for at least 24 hours in 4% paraformaldehyde in 10 mm PB. To visualize biocytin-filled cells, sections were incubated with streptavidin conjugated with Alexa Fluor 546 (1:1000; S11225, Thermo Fisher) overnight. The next day, sections were washed and incubated with thiodiethanol (TDE; 60% in 10 mm PBS, Sigma-Aldrich) for 20 min and coverslipped with TDE. Images of biocytin-filled neurons were acquired with Axio Imager M2 coupled with Apotome-2 (Zeiss).

### Analyses and statistics

Analyses were performed offline with the software IGOR (Wavemetrics) and Clampfit 10 (Molecular Devices). Values are expressed as means ± SE. All cells with stable resting potentials that generated overshooting spikes were included in the analyses. No data were excluded. All statistical tests are two-sided. We used χ^2^ tests to compare the incidence of particular properties in different samples. Paired or unpaired *t* tests, as appropriate, were used to assess significance of differences between different samples with a significance threshold of *p* = 0.05. We also used a mixed-effect ANOVA to compare current-evoked spiking in shell and core neurons.

## Results

A total of 159 VMH neurons (core, *n* = 116; shell, *n* = 43) were recorded in Vgat-ires-Cre-Ai6 mice (*n* = 30) or Vglut2-ires-Cre-Ai6 mice (*n* = 7). Because different parts of the VMH core play different roles (Lin et al., 2011; Silva et al., 2013; Lee et al., 2014; Wang et al., 2015; Sakurai et al., 2016)—that is, mediate different behaviors in response to distinct stimuli—core and shell neurons were further subdivided based on their location (core-DM, *n* = 57; core-VL, *n* = 59; shell-DM, *n* = 23, shell-VL, *n* = 20). The morphologic properties of an additional subset of shell (*n* = 8) and core (*n* = 6) neurons were revealed by including biocytin (0.75%) in the pipette solution.

### Influence of shell neurons on core cells

GABAergic and glutamatergic neurons are differentially distributed in VMH’s shell and core regions (Fig. 1*C*,*D*). As previously reported (Hashikawa et al., 2017), the core region displayed a high concentration of glutamatergic cells (Fig. 1*C*) but very few GABAergic neurons (Fig. 1*D*). In the core, the ratio of Vglut2^+^ to Vgat^+^ cells was 23.13 ± 1.75, whereas in the shell, it was 1.52 ± 0.1 (see Methods). This difference resulted from the fact that the concentration of glutamatergic cells was much lower in the shell (80.01 ± 8.86/mm^3^) than the core (210.31 ± 26.76/mm^3^), whereas the concentration of GABAergic cells was nearly five times higher in the shell (69.86 ± 8.14mm^3^) than core (15.81 ± 0.96/mm^3^). It should be noted that GABAergic and glutamatergic neurons were distributed differently in the shell. Whereas GABA cells were distributed homogeneously in the shell, glutamatergic cells generally occurred in small but dense clusters that correspond to the “cell bridges” described in Fig. 2.

**Figure 3.**
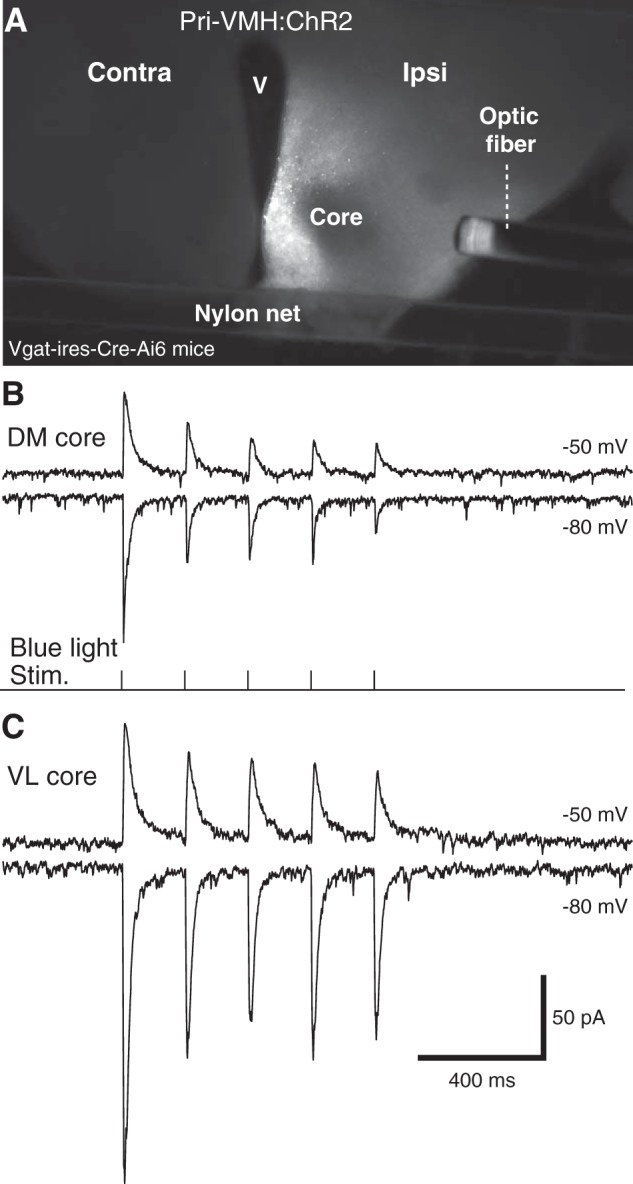
GABAergic cells of the VMH’s shell inhibit core neurons. ***A***, Distribution of mCherry reporter in the hypothalamus following infusion of AAV-EF1α-DIO-hChR2-mCherry just outside the core region. Because the virus was infused in Vgat-ires-Cre-Ai6 mice, ChR2 expression was restricted to Cre-expressing GABAergic neurons. Note absence of fluorescence in core region. ***B***, ***C***, Examples of IPSCs evoked in DM (***B***) and VL (***C***) core neurons at holding potentials of –50 (top trace) and –80 mV (second trace) by blue light stimuli (bottom trace of ***B***). Contra, contralateral; ipsi, ipsilateral; V, ventricle.

We first tested the hypothesis that GABAergic neurons in the shell and immediately surrounding region contribute inhibitory synapses onto core neurons (Murphy and Renaud, 1968; Fu and van den Pol, 2008). The contribution of glutamatergic shell neurons was not investigated in the present study. In Vgat-ires-Cre-Ai6 mice (*n* = 8), we infused AAV-EF1α-DIO-hChR2-mCherry just outside the core region, thus restricting expression of ChR2 to Cre-expressing GABAergic neurons (Fig. 3*A*). In support of this hypothesis, blue light stimuli reliably elicited IPSCs in all tested core neurons (DM, 70.01 ± 19.54 pA, *n* = 9, Fig. 3*B*; VL, 79.37 ± 11.31 pA, *n* = 8, Fig. 3*C*) with no significant difference between cells recorded in the DM and VL sectors (unpaired *t* test, *t* = 0.43, *p* = 0.68). These IPSCs reversed at around –60 (–63.0 ± 1.6 mV), were monophasic, and could follow trains of blue light stimuli at 5 Hz, albeit with marked attenuation from the first to the following stimuli.

**Figure 4.**
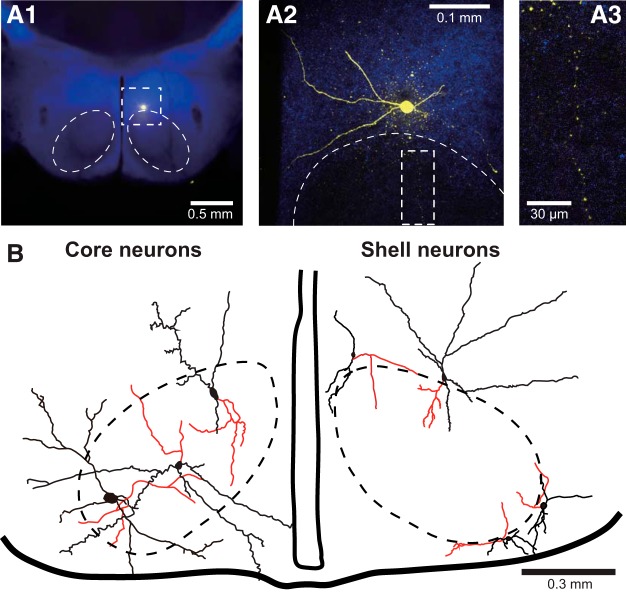
Morphologic properties of shell and core neurons. ***A***, Example of biocytin-filled shell neuron (yellow pseudocolor) shown at a low (***1***) and high (***2***) magnification. The region enclosed in a dashed rectangle in ***A2*** is shown at a higher magnification in ***A3***, revealing that this shell neuron contributes a varicose axon into the core region. ***B***, Drawings of three core (left) and four shell (right) neurons. Red lines represent axons.

Further evidence in support of the notion that GABAergic shell neurons provide inhibitory inputs to core neurons was obtained by revealing their morphologic properties with biocytin. As shown in Fig. 4, all recovered Vgat+ shell neurons (*n* = 8) contributed varicose axons into the core region as well as in the shell. Moreover, all the core neurons we recovered (*n* = 6) had dendritic branches extending into the shell and beyond.

### Transmitter used by BM and BNSTa axons ending in the VMH

**Figure 5.**
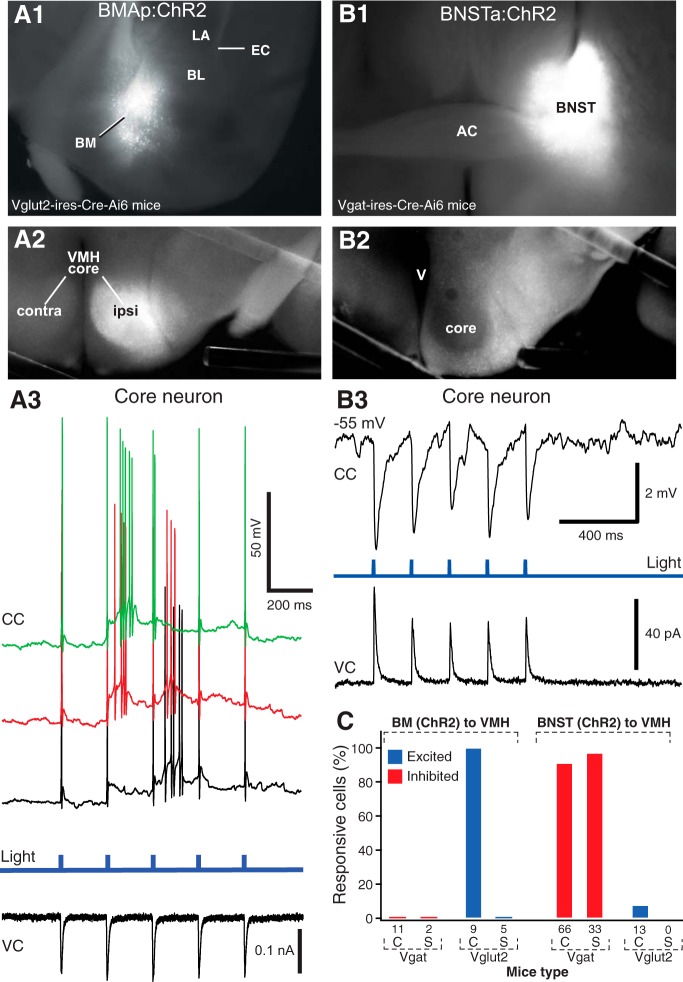
Most VMH-projecting BM and BNSTa neurons are glutamatergic and GABAergic, respectively. The virus AAV-EF1α-DIO-hChR2-mCherry was infused in BM (***A1***) or BNSTa (***B1***) of Vglut-ires-Cre-Ai6 or Vgat-ires-Cre-Ai6 mice, respectively. This resulted in pronounced mCherry reporter expression in the VMH core (***A2***) or shell (***B2***), respectively. ***A3***, In Vglut2-ires-Cre-Ai6 mice that received virus infusions in BM, blue light stimuli (fourth trace) elicited suprathreshold EPSPs in core neurons [black, red, and green lines represent different current-clamp (CC) trials] while the cell was at rest. A voltage-clamp (VC) recording in the same cell and testing conditions are shown at the bottom of ***A3***. ***B3***, In Vgat-ires-Cre-Ai6 mice that received virus infusions in BNST, blue light stimuli (second trace) elicited IPSPs (top trace) and IPSCs (bottom trace) in core neurons. ***C***, Proportion of cells (C, core neurons; S, shell neurons) responsive to blue light stimuli (blue, excited; red inhibited) following infusion of AAV-EF1α-DIO-hChR2-mCherry in BM (left) or BNSTa (right) and depending on whether the infused mice were Vglut-ires-Cre-Ai6 mice or Vgat-ires-Cre-Ai6 mice (bottom). Number of tested cells is indicated by the numerals just below the bars.

To the best of our knowledge, the identity of the neurotransmitters used by VMH-projecting BM and BNSTa neurons has not been ascertained. However, as detailed in the Discussion, there is much indirect evidence suggesting that they are glutamatergic and GABAergic, respectively. To settle this question, we took advantage of the selective expression of Cre-recombinase by glutamatergic or GABAergic neurons in Vglut2-ires-Cre-Ai6 or Vgat-ires-Cre-Ai6 mice (respectively) to restrict the expression of ChR2 and the reporter mCherry to either cell type (Fig. 5*A*,*B*).

Following BM infusions of AAV-EF1α-DIO-hChR2-mCherry in Vgat-ires-Cre-Ai6 mice (*n* = 3), no anterogradely labeled axons could be observed in the VMH, and blue light stimuli elicited no synaptic responses in 6 DM and 5 VL core neurons (Fig. 5*C*). In contrast, the same virus infusions in the BM of Vglut2-ires-Cre-Ai6 mice (Fig. 5*A1* ; *n* = 4) led to high reporter expression throughout the core of VMH (Fig. 5*A2* ), and blue light stimuli elicited suprathreshold EPSPs from rest in all tested core neurons (Fig. 5*A3* ; DM, *n* = 5; VL, *n* = 4) but no response in Vglut2-negative shell neurons (*n* = 5; Fig. 5*C*).

An inverse pattern of results was obtained following infusions of the same virus in BNSTa. That is, in Vglut2-ires-Cre-Ai6 mice (*n* = 2), very little mCherry expression could be detected in the shell or core of VMH, and blue light stimuli generally elicited no response in core neurons (DM, *n* = 6; VL, *n* = 7; Fig. 5*C*). Only one of the tested cells displayed a response, and it consisted of low-amplitude (∼2-mV) subthreshold EPSPs. In contrast, following the same virus infusion in the BNSTa of Vgat-ires-Cre-Ai6 mice (Fig. 5*B1* ; *n* = 19), pronounced mCherry expression was seen in the shell of VMH and surrounding region (Fig. 5*B2* ). Moreover, blue light stimuli elicited IPSPs in most core (91% of 66; Fig. 5*B3* ) and all Vgat positive shell (100% of 33) neurons (Fig. 5*C*).

### Comparison between the impact of BNSTa inputs on VMH core and shell neurons

Overall, the above experiments support the conclusion that most (if not all) VMH-projecting BM neurons are glutamatergic, whereas GABAergic cells constitute the prevalent type of BNSTa neurons targeting the VMH region. These tests also revealed that although BNSTa axons do not end in the VMH’s core, they nonetheless form inhibitory synapses with core neurons, likely on their distal dendrites in the shell region (Millhouse, 1973a,b; Fu and van den Pol, 2008). Thus, BNSTa inputs can influence core neurons in two ways: via a direct inhibition and indirectly, through the inhibition of shell neurons (disinhibition).

**Figure 6.**
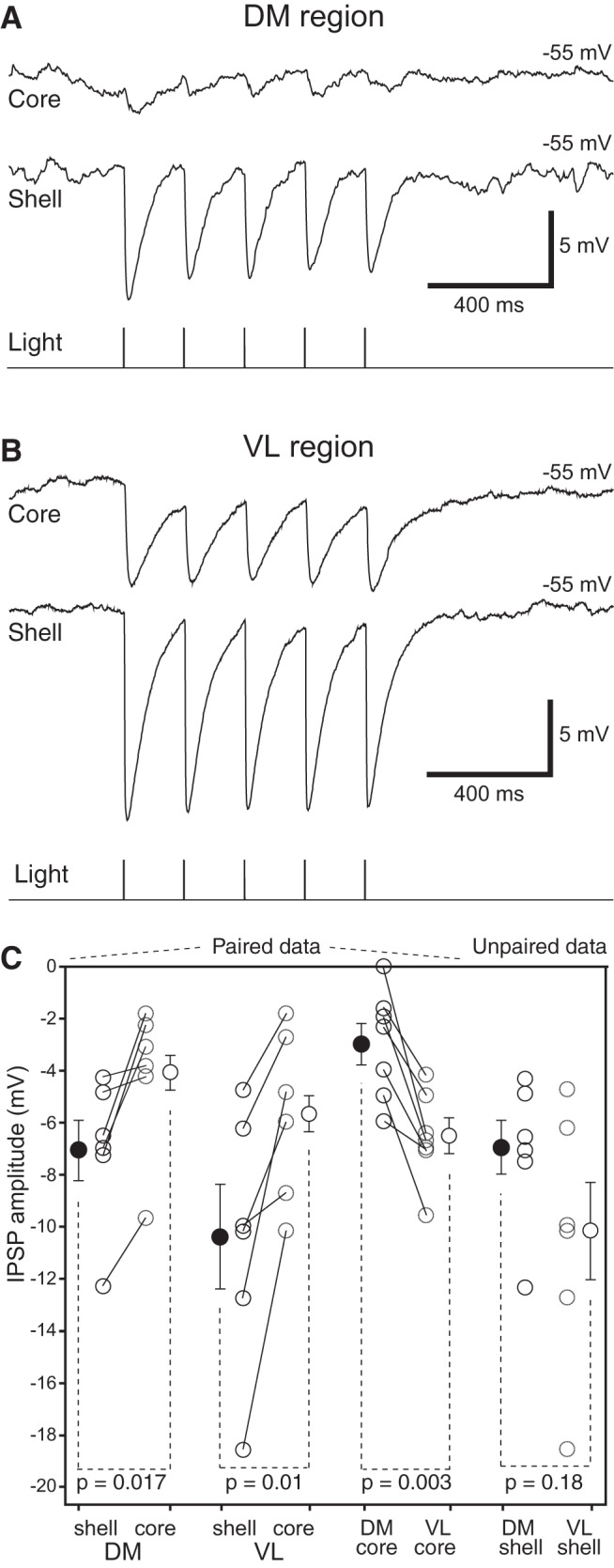
Contrasting influence of BNSTa inputs on shell and core neurons in different VMH sectors. The virus AAV-EF1α-DIO-hChR2-mCherry was infused in BNSTa of Vgat-ires-Cre-Ai6 mice. Blue light stimuli elicited higher-amplitude IPSPs in shell than core neurons whether they were recorded in the DM (***A***) or VL (***B***) regions. (**C**) Average ± SEM IPSP amplitude from –55 mV following light-induced activation of BNSTa axons in the cell types and regions indicated at bottom. Circles connected by lines indicate individual experiments. Isolated circles are group averages.

To determine the relative importance of these two modes of action, we first compared the amplitude and duration of the IPSPs seen in shell and core neurons following blue light stimulation of BNSTa axons in Vgat-ires-Cre-Ai6 mice (Fig. 6). To control for variations in the extent of infection between mice, multiple Vgat-positive shell and Vgat-negative core neurons were recorded in each mouse, and the data were averaged. Statistical comparisons were performed at the mouse level, using these averages. Whether the recordings were performed in the DM (Fig. 6*A*; 19 shell and 14 core neurons recorded in 6 mice) or VL (Fig. 6*B*; 14 shell and 17 core neurons recorded in 6 mice) sectors, blue light stimulation of BNSTa axons elicited significantly larger IPSPs in shell than core neurons (Fig. 6*C*; DM core –4.11 ± 1.16 mV, DM shell –7.06 ± 1.16 mV, *t* = 3.543, *p* = 0.017; VL core –5.66 ± 1.34 mV, VL shell –10.36 ± 2.02 mV, *t* = 4.052, *p* = 0.01).

Of note, although BNSTa synapses ending in the distal dendrites of core neurons and shell neurons are electrotonically more compact than core neurons (see below), their extrapolated reversal IPSP potential (core –66.2 ± 1.5 mV, *n* = 26; shell –69.1 ± 1.8 mV, *n* = 14; *t* test, *t* = 1.217; *p* = 0.231) and time course (10%–90% rise time: core, *n* = 53, 10.6 ± 0.8 ms; shell, *n* = 32, 9.8 ± 1.2 ms, *t* = 0.609, *p* = 0.544; duration at half-amplitude: core, 93.5 ± 5.6 ms, shell, 89.2 ± 6.7 ms, *t* = 0.481, *p* = 0.632) did not differ.

While IPSP amplitudes were nearly twice as high in shell than core neurons of the DM and VL sectors, neurons recorded in the VL sector, whether they were shell or core neurons, displayed IPSPs of higher amplitude than their counterparts in the DM sector. This aspect was further studied systematically in seven Vgat-ires-Cre-Ai6 mice where we recorded at least one DM and one VL core neuron (total of 17 and 18, respectively) in each mouse. In this dataset, IPSP amplitudes were more than twice as high in VL (–6.53 ± 0.65 mV) as DM (–2.99 ± 0.79 mV) core neurons (paired *t* test, *t* = 4.84, *p* = 0.003; Fig. 6C).

### Electroresponsive properties of core and shell neurons

The above experiments indicate that in the quiescent conditions of brain slices kept *in vitro*, BNSTa axons exert a stronger inhibitory influence over shell than core neurons, suggesting that BNSTa-to-VMH connections favor disinhibition over inhibition of core neurons. However, expression of this bias will depend on several factors, including the firing rate of shell neurons. That is, depending on whether GABAergic shell neurons fire at high or low rates, core neurons will experience more or less disinhibition. While the artificial conditions of brain slices prevent us from addressing this question, they allow us to study a major determinant of firing rates, the cell’s electroresponsive properties.

To investigate this aspect, we delivered graded series of depolarizing and hyperpolarizing current pulses to shell (*n* = 41) and core (*n* = 97) VMH neurons from various membrane potentials. From the cells’ voltage response to the –20-pA current pulses, we derived their input resistance and time constant. We also assessed their current-evoked and spontaneous discharge patterns. Although these tests were conducted in the DM and VL sectors, the data are pooled below because shell and core neurons displayed a similar range of properties irrespective of their location.

As detailed in Table 1, the passive properties of shell and core neurons differed significantly. Shell neurons had a markedly higher input resistance and a slightly more depolarized resting potential than core neurons. Also, shell neurons generated action potentials of significantly lower amplitude than core neurons, but spike threshold and duration did not differ significantly. Last, a similar proportion of shell (37% or 15 of 41) and core (33% or 32 of 97) neurons fired spontaneously at rest (χ^2^ = 0.237; *p* = 0.627). Among these spontaneously active cells, firing rates were 64% higher in shell (2.67 ± 0.71 Hz) than core neurons (1.63 ± 0.38 Hz), albeit not significantly so (*t* test, *t* = 1.42, *p* = 0.162).

**Table 1.** Physiologic properties of core and shell neurons

Neuron	Resting *V_*m*_* (mV)	Rin (MΩ)	AP height (mV)	AP threshold (mV)	AP half-width (ms)	Time constant (ms)
Core (*n* = 97)	–58.6 ± 0.6	722.8 ± 25.9	99.0 ± 0.9	–43.1 ± 0.3	0.76 ± 0.03	40.3 ± 1.6
Shell (*n* = 41)	–56.5 ± 0.9	1028.8 ± 64.9	93.4 ± 1.5	–42.0 ± 0.7	0.71 ± 0.03	41.6 ± 2.4
*p* value	0.049	<0.001	0.0013	0.119	0.064	0.65
*t* value	–1.984	–5.278	3.303	–1.567	1.865	0.452

**Figure 7.**
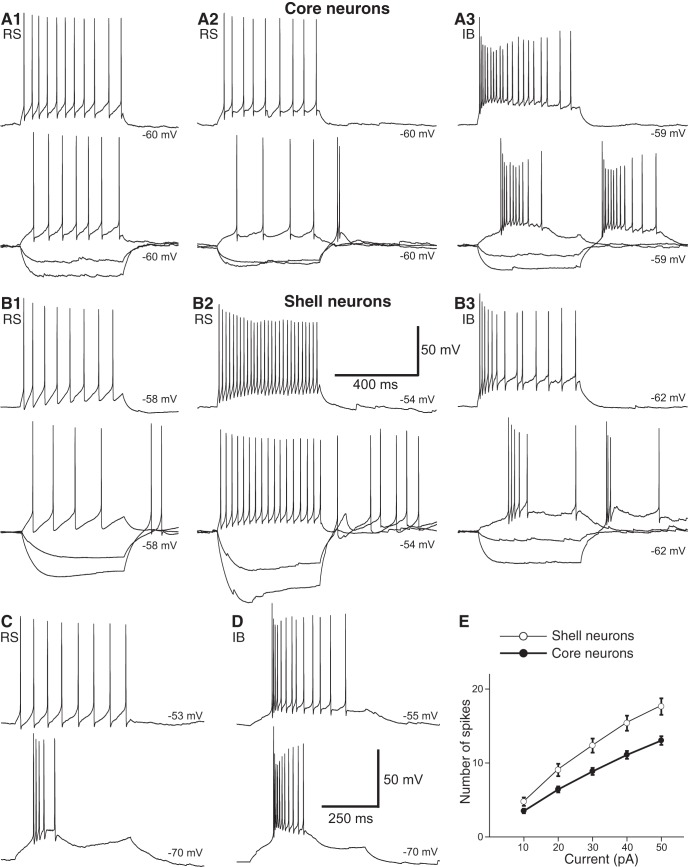
Electroresponsive properties of VMH neurons. ***A***, ***B***, Voltage responses of six different core (***A***) and shell (***B***) neurons to negative (–20 and –40 pA) and positive (20 and 40 pA) current pulses from rest (numerals to the right of the traces). Top trace in ***A1–3*** and ***B1–3*** was offset for clarity. ***C***, ***D***, Effect of changes in membrane potential (numbers to the right) on the firing pattern of RS (***C***) and IB (***D***) neurons. In both cases, a current pulse of 20 pA was applied at the negative membrane potential and 10 pA at the more positive membrane potential. ***E***, Number of current-evoked action potentials (*y*-axis; average ± SEM) plotted as a function of injected current (*x*-axis; 500-ms pulses). Calibration bars in B2 apply to panels ***A1–3*** and ***B1–3***. Calibration bars in ***D*** also apply to ***C***.

As to the dynamics of current-evoked firing, there was much heterogeneity in both cell types. Based on the cells’ firing patterns to depolarizing current pulses applied from rest, two main types of core neurons could be distinguished, regular spiking (RS; 56%; Fig. 7*A1*,*A2* ) and intrinsically bursting (IB; 44%; Fig. 7*A3* ), both of which could express post-anodal bursting (67% of RS and 70% of IB) or not (33% of RS and 30% of IB). Although RS (Fig. 7*B1*,*B2* ) and IB (Fig. 7*B3* ) neurons were also observed among shell neurons, RS cells accounted for a significantly higher proportion of shell (76%) than core neurons (57%) neurons (χ^2^ = 4.388; *p* = 0.036).

In core and shell RS neurons that lacked a rebound burst at the end of negative current pulses, membrane hyperpolarization failed to transform their depolarization-evoked tonic firing into spike bursts. In contrast, reminiscent of thalamic relay cells (Llinás and Jahnsen, 1982), in those cells with a clear rebound burst, membrane hyperpolarization transformed their depolarization-evoked tonic discharges into low-threshold spike bursts (Fig. 7*C*). As to IB neurons, membrane depolarization did not transform their spike bursts into tonic discharges, although it did cause single spikes to occur after the initial spike burst (Fig. 7*D*).

Since RS cells accounted for the majority of neurons in both VMH subsectors, we compared current-evoked spiking in core versus shell RS neurons using 500-ms current pulses ranging between 10 and 50 pA in amplitude and applied at rest. Consistent with the fact that shell neurons had a higher input resistance than core neurons (Table 1), they generated significantly more action potentials (Fig. 7*E*; two-way mixed effect ANOVA, *F*_between(1,84)_ = 12.7, *p* < 0.001). While there was no difference in this respect between IB cells of the shell and core (two-way mixed effect ANOVA *F*_between_(1,50) = 0.03, *p* = 0.86), the same comparison between all shell and core neurons, that is including both RS and IB cells, remained significant (two-way mixed effect ANOVA *F*_between_(1136) = 6.04, *p* = 0.015).

## Discussion

The present study examined the influence of BM and BNSTa projections to VMH neurons. Although BM is the main source of non-olfactory information about predators and aggressive conspecifics to the VMH, it can also influence it indirectly through its projections to BNSTa. However, most BNSTa neurons are GABAergic, and it seems paradoxical that BNSTa neurons, after being recruited by BM, would counter BM’s excitatory effects by inhibiting VMH neurons. In a likely solution to this paradox, our data suggest that BM and BNSTa inputs can actually influence VMH’s projection cells in a synergistic manner, the former through excitation and the latter through disinhibition.

### Transmitter used by VMH-projecting BM and BNSTa neurons

Before the present study, the neurotransmitter used by VMH-projecting BM and BNSTa neurons had not been formally identified. However, much indirect evidence suggested that they use glutamate and GABA, respectively. In the case of BM, it was found that anterogradely labeled axon terminals from different nuclei of the basolateral amygdaloid complex (of which BM is a part) are enriched in glutamate and form asymmetric synapses with cortical and central amygdala neurons (Smith and Paré, 1994; Paré et al., 1995). However, one study reported that some GABAergic neurons of the basolateral amygdala have extrinsic projections (McDonald et al., 2012). As to BNSTa, *in situ* hybridization studies reported that the vast majority of BNSTa neurons are GABAergic (Day et al., 1999; Poulin et al., 2009). Nevertheless, although there are very few glutamatergic neurons in BNSTa, it remained possible that they project to the VMH.

Here we addressed this question by taking advantage of the selective expression of Cre-recombinase in glutamatergic or GABAergic neurons in two mouse lines, allowing us to restrict ChR2 expression to either cell type. Using this approach, we found that most (if not all) VMH-projecting BM neurons use glutamate as a transmitter. No evidence of a GABAergic innervation of VMH by BM was detected. That is, following BM infusions of AAV-EF1α-DIO-hChR2-mCherry in Vgat-ires-Cre-Ai6 mice, no anterograde labeling was observed in the VMH. Conversely, in BNSTa experiments, evidence of a robust GABAergic projection was obtained. In this case however, evidence of a minor glutamatergic contingent was observed.

In support of these findings, there are precedents in the literature for the contribution of GABAergic and glutamatergic neurons to the projections of BNSTa. For instance, glutamatergic and GABAergic neurons both project to other BNST sectors (Turesson et al., 2013), to the ventral tegmental area (Kudo et al., 2012), and to the central nucleus of the amygdala (Gungor et al., 2015). In the latter two cases, however, most of the projections are inhibitory, as we saw in the VMH. An important question to be addressed in future studies will be to determine if GABAergic and glutamatergic BNSTa cells contact different subtypes of VMH neurons.

### Interaction between BM and BNSTa projections to the VMH

In addition to using different neurotransmitters, BM and BNSTa send non-overlapping projections to the VMH. BM projects to the VMH’s core (Dong and Swanson, 2006), where glutamatergic projections cells are found (Fu and van den Pol, 2008), whereas BNSTa targets the VMH’s shell and surrounding region (Dong and Swanson, 2006), where GABAergic cells are concentrated (Fu and van den Pol, 2008). Since BM contributes a very strong glutamatergic projection to BNSTa (Krettek and Price, 1978; Dong et al., 2001; Nagy and Paré, 2008), VMH-projecting BM and BNSTa neurons are expected to be activated in parallel. This raises the question of how the joint activation of BM and BNSTa inputs affects VMH’s output neurons.

To address this question, we compared their impact on glutamatergic core and GABAergic shell neurons using optogenetic methods. While activation of glutamatergic BM inputs fired most core cells, shell neurons remained unresponsive. In contrast, activation of GABAergic BNSTa inputs elicited IPSPs in both core and shell neurons. Since these IPSPs had a markedly higher amplitude in shell than core neurons, the net influence of BNSTa on core neurons appears to be a disinhibition. However, because BNSTa inputs directly inhibit core neurons, their influence will depend on the status of shell neurons. That is, the impact of BNSTa inputs could shift from a disinhibition of VMH’s output cells, when shell neurons fire at high rates, to an inhibition of core neurons, when shell neurons are inactive.

While we are not aware of unit recording studies on the firing rates of shell neurons in behaving animals, we note that their electroresponsive properties predispose them to display elevated activity levels. These properties include a high input resistance, a relatively depolarized resting potential, and the ability to sustain high firing rates with modest spike frequency adaptation. In any event, it is clear that the parallel regulation of VMH by BM and BNSTa imparts flexibility to this innate defensive network. The presence of a small population of glutamatergic shell neurons, whose connectivity was not investigated in the present study, might further enhance this flexibility.

### *Relation between BNST*a *and VMH activity in the genesis of defensive behaviors*

Like VMH, BNSTa has been implicated in the genesis of defensive behaviors (Gungor and Paré, 2016) and is commonly believed to mediate long-lasting states of increased vigilance and apprehension in the anticipation of ill-defined and unpredictable perils (Walker et al., 2009). For instance, BNSTa lesion or inactivation interferes with anxiety-like responses to alarm pheromones (Breitfeld et al., 2015), predator odors (Fendt et al., 2003; Xu et al., 2012), and bright lights (Walker and Davis, 1997). Moreover, exploratory behavior in assays that measure fear of open spaces, such as the elevated plus maze, also depends on BNSTa activity (Waddell et al., 2006; Duvarci et al., 2009; Kim et al., 2013). While BNSTa projections to the paraventricular hypothalamic nucleus (Sawchenko and Swanson, 1983; Moga and Saper, 1994) and brainstem nuclei (Holstege et al., 1985; Gray and Magnuson, 1987) such as the ventrolateral periaqueductal gray are commonly thought to mediate BNSTa’s influence over defensive behaviors, the present findings suggest an additional mechanism, namely the disinhibition of VMH’s core neurons. An important challenge for future studies will be to test this possibility.

## Synthesis

Reviewing Editor: Menno Witter, Norwegian University of Science and Technology Decisions are customarily a result of the Reviewing Editor and the peer reviewers coming together and discussing their recommendations until a consensus is reached. When revisions are invited, a fact-based synthesis statement explaining their decision and outlining what is needed to prepare a revision will be listed below. The following reviewer(s) agreed to reveal their identity: NONE. Note: If this manuscript was transferred from JNeurosci and a decision was made to accept the manuscript without peer review, a brief statement to this effect will instead be what is listed below.

The authors have adequately addressed the comments of both reviewers of the now transferred manuscript. The manuscript contributes important data in the network related to VMH and the conclusions are properly supported by experimental data.

I request that the authors provide a final version of the manuscript, without marking changes and make sure that the paper is formatted according to the requirements of eNeuro
